# Identifying Hub Genes for Heat Tolerance in Water Buffalo (*Bubalus bubalis*) Using Transcriptome Data

**DOI:** 10.3389/fgene.2019.00209

**Published:** 2019-03-13

**Authors:** Shenhe Liu, Tingzhu Ye, Zipeng Li, Jun Li, Ahmad Muhammad Jamil, Yang Zhou, Guohua Hua, Aixin Liang, Tingxian Deng, Liguo Yang

**Affiliations:** ^1^Ministry of Education, Key Laboratory of Agricultural Animal Genetics, Breeding and Reproduction, College of Animal Science and Technology, Huazhong Agricultural University, Wuhan, China; ^2^Department of Immunology, Zunyi Medical College, Zunyi, China; ^3^Guangxi Provincial Key Laboratory of Buffalo Genetics, Breeding and Reproduction Technology, Buffalo Research Institute, Chinese Academy of Agricultural Sciences, Nanning, China

**Keywords:** buffalo, heat tolerant, hub gene, miRNA analysis, transcriptome analysis, WGCNA

## Abstract

Heat stress has a detrimental effect on the physiological and production performance of buffaloes. Elucidating the underlying mechanisms of heat stress is challenging, therefore identifying candidate genes is urgent and necessary. We evaluated the response of buffaloes (*n* = 30) to heat stress using the physiological parameters, ELISA indexes, and hematological parameters. We then performed mRNA and microRNA (miRNA) expression profiles analysis between heat tolerant (HT, *n* = 4) and non-heat tolerant (NHT, *n* = 4) buffaloes, as well as the specific modules, significant genes, and miRNAs related to the heat tolerance identified using the weighted gene co-expression network analysis (WGCNA). The results indicated that the buffaloes in HT had a significantly lower rectal temperature (RT) and respiratory rate (RR) and displayed a higher plasma heat shock protein (HSP70 and HSP90) and cortisol (COR) levels than those of NHT buffaloes. Differentially expressed analysis revealed a total of 753 differentially expressed genes (DEGs) and 16 differentially expressed miRNAs (DEmiRNAs) were identified between HT and NHT. Using the WGCNA analysis, these DEGs assigned into 5 modules, 4 of which were significantly correlation with the heat stress indexes. Interestingly, 158 DEGs associated with heat tolerance in the turquoise module were identified, 35 of which were found within the protein-protein interaction network. Several hub genes (*IL18RAP*, *IL6R*, *CCR1*, *PPBP*, *IL1B*, and *IL1R1*) were identified that significantly enriched in the Cytokine-cytokine receptor interaction. The findings may help further elucidate the underlying mechanisms of heat tolerance in buffaloes.

## Introduction

Heat stress is a multi-factorial problem that results in the huge economic losses for many livestock enterprises across the world (Ferreira et al., 2016), particularly in the dairy industry with an estimated 897–1500 million dollars in annual economic losses (St-Pierre et al., 2003). Water buffalo (*Bubalus bubalis*) serve as the important dairy livestock that provides more than 5% of the world’s milk supply (Jun Jing et al., 2013). These animals are generally healthy animals that govern the agricultural economy of several countries and are typically adjusted to the hot/humid climates compared to other dairy animals. However, they still feel great distress if exposed to direct solar radiation or working in the sunlight during hot weather, thereby affecting their production performance. For example, increased rectal temperature (RT) and respiratory rate (RR) result in a reduction of milk production and poor fertility in buffaloes (Vale, 2010; Maf, 2017; Shenhe et al., 2018). Three mitigation strategies can be recently used for combating the adverse effects of summer heat stress in animals, such as physical modification of the environment (Fournel et al., 2017), development of genetically heat tolerant breeds (Breede and Collier, 1986), and nutritional modification (West, 1999). Notably, breeding for heat tolerance was the most cost-effective measure for mitigating heat stress (Renaudeau et al., 2012). A useful measure to genetically dissect the traits associated with heat stress in livestock can be utilized for cultivating breeds. However, little information on the identification of candidate genes related to heat stress in buffaloes has been reported. Therefore, identifying the candidate genes for the traits of interest is a feasible strategy that helps in the understanding of biological function of these genes affecting productive performance in livestock.

Transcriptome sequencing is a vital platform that encompasses a wide variety of applications from simple mRNA profiling to the identification of non-coding RNA (ncRNA). RNA Sequencing (RNA-Seq) was initially developed the approach to transcriptome profiling that can generate lists of expressed genes in specific tissues to ultimately detect differentially expressed genes (DEGs) (Wang et al., 2009). It has been widely used for exploring DEGs associated with complex traits, such as milk production (Seo et al., 2016), reproduction (Han Ying et al., 2015), meat quality (Jung et al., 2012), and coat color (Li et al., 2012). Transcriptome data has also been utilized for constructing the gene co-expression network (GCN), aiming to identify the associated genes. GCN is an undirected graph that consists of genes (nodes) connected to other genes by edges. GCN analysis can improve the power of gene detection and provide new insights into the complex traits and diseases by grouping genes into modules that are enriched for particular biological processes (Chen et al., 2016). Interestingly, Weighted Gene Co-expression Network Analysis (WGCNA) method as a network approach, was widely used for detecting highly co-expressed gene set (Zhang and Horvath, 2005). Also WGCNA method can group genes into the specified modules based on the high correlations between co-expression genes across the samples, resulting in a cluster of genes that share a similar function (Langfelder and Horvath, 2008; Atila et al., 2009). Cumulative studies on the transcriptome data using the WGCNA method to investigate complex traits or disease in animals, such as obesity (Kogelman et al., 2014), residual feed intake (Kong et al., 2016), and fat deposition (Oliveira et al., 2018), and in human, including brain evolution (Oldham et al., 2006), schizophrenia (Chang et al., 2017), autism (Gupta et al., 2014), neuroblastoma (Yang et al., 2018b), and eating disorders (Yang et al., 2018a) has been reported. However, RNA-Seq has not been used to identify the candidate genes related to heat tolerance in buffaloes.

Another advantage of transcriptome data is the use for discovery of ncRNA, including MicroRNAs (miRNAs), Long non-coding RNAs (LncRNA), and Circular RNA (circRNA). The miRNAs are ∼22nt small ncRNAs that play critical roles in various biological processes via regulation of gene expression and can adversely affect the post-transcriptional mRNA stability or translation (Bushati and Cohen, 2007). Several studies have revealed that some miRNAs involved in complex post-transcriptional regulatory mechanisms response to heat stress in different animals, such as rodents (Islam et al., 2013), fish (Zhang et al., 2017), and cattle (Zheng et al., 2014; Sengar et al., 2017). For instance, let-7d miRNA is involved in response to heat stress in rat small intestine (Yu et al., 2011) and fish (Zhang et al., 2017). MiR-145 and miR-125 have also been reported to be involved in cell responses to heat stress (Leung and Sharp, 2010; Zhang et al., 2017). However, little information was available about miRNA expression patterns of heat tolerant in buffaloes.

In this study, we performed the transcriptome analysis of buffalo blood samples in response to heat stress, aiming to identify the DEGs between heat tolerant (HT) and non-heat tolerant (NHT) buffaloes. The miRNA-Seq analysis was also used for discovery and analysis of miRNA in response to heat stress between HT and NHT buffaloes. Further, WGCNA was conducted to investigate the DEGs associated with heat tolerance. Finally, we identified the hub gene related to heat tolerance and constructed the mRNA-miRNA interaction network. These genes and their interaction network will contribute to a better understanding of the genetic mechanisms underlying the heat tolerance in buffaloes.

## Materials and Methods

### Experiment Design and Sample Collection

A total of 30 healthy crossbred female buffaloes (Nili-Ravi × Murrah) between 3rd and 4th parity, weighing approximately 562 ± 16.2 kg, were selected for this study. Roughage, concentrate supplements, and clean water was fed at libitum.

We used heat stress indexes to evaluate the response of buffaloes (*n* = 30) to heat stress, including physiological parameters, ELISA indexes, and hematological parameters. First, two physiological indicators, including RT and RR were taken at 1:00–3:00 pm for 5 consecutive days in August. RT and RR were measured according to the methods (Shenhe et al., 2018). Next, a single blood sample in duplicate for each buffalo was taken at 2:00 p.m. within a 5 days window for plasma separation and hematological examination. Blood samples were centrifuged (3000 g for 15 min) to separate the plasma samples, and used to measure HSP70 (Mlbio, Shanghai, China), HSP90 (Mlbio, Shanghai, China), and cortisol (COR) levels (Mlbio, Shanghai, China) following the ELISA guidelines. All assays had intra- and inter-assay coefficients of variation of less than 10 and 15%, respectively. Moreover, the whole blood samples were used for testing hematological parameters such as hemoglobin (Hb), hematocrit (Hct) and red blood cells (RBCs) using Blood Routine Apparatus (Sysmex Shanghai Ltd., China). Temperature and humidity index (THI) was also calculated by the formula (Kendall and Webster, 2009): THI = (1.8 × AT + 32) - (0.55–0.0055 × RH) × (1.8 × AT - 26), where the ambient temperature (AT) and relative humidity (RH) were recorded from 7:00 a.m. to 7:00 p.m., once every 2 h, for 5 consecutive days in August.

To better ascertain NH and NHT individuals by using heat stress indexes, a total of 30 buffaloes was carried out to perform principal component analysis (PCA) and their result was displayed in Supplementary Figure S1. According to the PC1, these animals can be grouped into two groups (HT, *n* = 7; NHT, *n* = 8). Next, we selected a total of 8 buffaloes in the present study that were used for the mRNA and miRNA sequencing. For them, 4 buffaloes were the HT group because they had a closer distance among them; another 4 buffaloes were selected for NHT group using the similar principle. A blood sample for each buffalo (*n* = 8) was collected from the external jugular vein at 2:30 p.m. in another day of August (THI = 87), and immediately placed in a non-RNA-enzyme tube containing Trizol reagent (Invitrogen, United States). Studies have reported heat stress in buffaloes begin at THI 75 (Dash et al., 2015); thus, our targeted THI of 87 in August, for this study is well within the range for identifying heat stressed animals.

### Transcriptome Profiling and Small RNA Analysis

For the RNA-Seq, total RNA from each sample was isolated using an RNA isolation kit (Tiangen, Beijing, China) and purified using a TruSeq RNA Sample Prep Kit V2 (Illumina Inc., San Diego, CA, United States) following the manufacturer’s protocols. Total RNA quality and quantity were determined by Agilent Bioanalyzer 2100 system (Agilent, Santa Clara, CA, United States) and SDS-PAGE, respectively. The cDNA library for each sample was constructed using the Illumina TruSeq^TM^ RNA Sample Preparation Kit (Illumina, San Diego, CA, United States). Overall, the poly (A) mRNAs were isolated from the 5 μg of total RNA using Oligo (dT) magnetic beads (Invitrogen, United States). The cDNAs were purified and amplified by PCR, followed by chemically fragmenting to ∼ 200 nt fragments and enriching with PCR to create the final cDNA libraries. The sequencing of eight cDNA libraries was performed by Illumina HiSeq^TM^ 2500 platform (Illumina, United States).

For the small RNA sequencing, small RNA Sample Prep Kit (Illumina, United States) was used to construct the library from 10 μg of total RNA. Briefly, small RNA fragments with the length of 18–30 nt were isolated and purified from total RNA for each sample by 15% denaturing polyacrylamide gel electrophoresis. Next, 3′ and 5′ RNA adaptors were ligated from the RNA pool using T4 RNA ligase, followed by the adaptor-ligated small RNAs subjected to RT-PCR amplification, cDNA synthesis, and PCR products purified using 10% PAGE to construct a small RNA library. Eight small RNA libraries were sequenced using Illumina HiSeq^TM^ 2500 sequencing platform (Illumina, United States). The raw data of mRNA and miRNA sequencing were deposited in the NCBI SRA database (BioProject ID: PRJNA517372).

### Analysis of Sequencing Data

The main steps and bioinformatics used for data analysis is shown in Supplementary Figure S2. For RNA-Seq data, the read quality of raw data was evaluated using the FastQC software (version 0.11.8)^1^ with the default settings, and clean reads were mapped against the reference genome (buffalo genome: *UOA_WB_1*) using HISAT2 ver. 2.1.0 (Kim et al., 2015). The StringTie ver. 1.3.5 (Pertea et al., 2016) software was used to detect the gene expression levels and normalize by library and gene length by calculating the Fragments Per Kilobase of Exon Per Million Fragments Mapped (FPKM) using the buffalo annotated file as a reference. The differential expression analysis between HT and NHT was performed using the DESeq2 (Love et al., 2014) and edgeR (Robinson et al., 2010) R-packages. The *P*-value ≤ 0.05 and Fold Change > 1.5 were defined as the cutoff criteria for the DEGs.

For the small RNA data, clean reads were generated by removing reads containing any of the following criteria: reads with 5′ adaptor contaminants, reads without 3′ adaptor, low-quality reads, reads without the insert tag, reads with poly-A, and reads shorter than 18 nt. After filtering, clean reads were aligned to the buffalo genome using the Bowtie ver. 1.1.2 (Langmead et al., 2009) with the defaults. The identification of mature and novel miRNAs was performed using the miRDeep2 (Friedländer et al., 2011). The mature sequences were downloaded from miRbase ver. 22.1^2^. The transcripts per million (TPM) values (miRNA total reads/total clean reads × 10^6^) were calculated by the normalized raw counts of miRNA reads using DESeq2. The differential analysis between HT and NHT was performed using the DESeq2 and edgeR. Only the miRNAs with Fold Change > 1.5 and *P*-value ≤ 0.05 were considered as differentially expressed miRNAs (DEmiNRAs). The 3′-UTR sequences of buffalo genes were obtained using the GenomicFeatures (Lawrence et al., 2013) R-package and TBtools ver. 0.665 (Chen et al., 2018). The miRanda ver.3.3a (Betel et al., 2008) was further used to predict the DEmiRNAs targets.

### Construction of Co-expression Modules

WGCNA (Langfelder and Horvath, 2008) R-package was used for the network construction of DEGs. First, we calculated the soft-thresholding power β using the pickSoftThreshold function of WGCNA. According to the scale-free topology criterion described by Zhang and Horvath (2005), β = 20 was chosen in this study because it results in a scale-free topology index (R2) of 0.90. Subsequently, the co-expression modules were constructed with the one-step network construction method using the blockwiseModules function of WGCNA. The dynamic hierarchical tree-cut algorithm was conducted with the following parameters: minModuleSize = 30, deepSplit = 3, mergeCutHeight = 0.25, and networkType = “signed hybrid.” Finally, the co-expression module structure was visualized using the plotDendroAndColors function of WGCNA.

### Identification of Modules-Heat Tolerance Relationships

The module-trait relationships were assessed by calculating the Pearson’s correlations between module eigengenes (MEs) and the heat stress indexes. ME was calculated by the first principal component, thereby obtaining the maximal amount of variation of the module.

### Hub Gene Analysis and mRNA-miRNA Network Construction

Hub genes, a few highly interconnected genes in a co-expression module, are thought to be biologically important. Module membership (MM) was defined as the correlation of the gene expression profile and MEs; Gene significance (GS) was defined as the absolute value of the correlation between the gene and traits of interest. Genes with high GS as well as high MM were chosen for downstream analysis. In the present study, the cutoff criteria of hub genes related to heat tolerance for DEGs modules were the following: MM > 0.8, GS > 0.2 and *P* ≤ 0.05 (Wang et al., 2017). For these selected hub genes, the protein-protein interaction (PPI) relationships were analyzed using STRING database^3^, with a confidence score > 0.4 and *P*-value ≤ 0.05 set as the cut-off threshold. Moreover, the relationship between hub genes and DEmiRNA targets was analyzed, and then their interaction network was visualized using the Cytoscape ver. 3.6 (Shannon et al., 2003).

### Functional Annotation

Potential function of DEGs, miRNA targets, module genes, and hub genes were annotated by the Gene Ontology (GO) functional analysis and the Kyoto Encyclopedia of Genes and Genomes (KEGG) pathway enrichment using the KEGG Orthology-Based Annotation System (KOBAS) 3.0 with cutoff criteria of *P* ≤ 0.05, aiming to identify their biological significance. The plot results were visualized using the ggplot2 (Wickham, 2016) R-package.

### Quantitative Real-Time PCR Confirmation of Hub Genes and DEmiRNAs

For validation of mRNA and miRNA sequencing expression results, 11 DEGs (including 6 hub genes) and 7 DEmiRNAs were randomly selected and analyzed by RT-qPCR. Primers were designed using Primer 5.0 software (Supplementary Table S1) and synthesized by Sangon Biotech (Shanghai) Co. Ltd. RevertAid First Strand cDNA Synthesis Kit (Thermo Fisher Scientific, United States) was used to reverse transcribe the total RNAs into cDNA following the manufacturer’s protocols for mRNA. Then qPCR was conducted using QuantiNova SYBR Green PCR Kit (QIAGEN, Shanghai, China). For miRNA, specific reverse transcription primers with step loop were synthesized and reverse transcription were performed using the RevertAid First Strand cDNA Synthesis Kit (Thermo Fisher Scientific, United States). The qPCR was completed using QuantiNova SYBER Green PCR Kit (QIAGEN, Shanghai, China). *GAPDH* gene and *U6* were used for normalizing the relative abundance of genes and miRNAs, respectively. The 2^-ΔΔCt^ method (Livak and Schmittgen, 2001) was used to analyze the data for all samples in triplicate technical replicates.

### Statistical Analysis

The physiological data, ELISA, hematological parameters and quantitative real-time PCR assay are expressed as mean ± standard error of the mean (SEM). Significant differences between samples were determined by Student’s *t*-test. Principal component analysis method (PRINCOMP Procedure) in SAS 9.4 was used to screen HT and NHT individuals. Differences were accepted as significant when adjusted *P* ≤ 0.05 (Bonferroni).

## Results

### Animal Source Description

**FIGURE 1:**
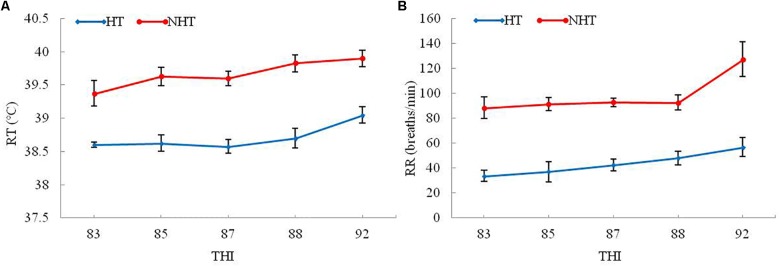
The RT and RR changes in HT and NHT buffaloes with the increase of THI. **(A)** The RT changes with the increase of THI. **(B)** The RR changes with the increase of THI.

To better ascertain NH and NHT individuals by using heat stress indexes, the PCA method was conducted to determine the sample’s exposure to heat stress, and their results were listed in Table 1. The results indicate that two physiological indicators including RT and RR in the HT group were significantly lower (*P* < 0.05) than those of NHT group, whereas the expression levels of plasma HSP70, HSP90, and COR were significantly higher (*P* < 0.05) compared to NHT group. However, there was no significant difference in hematological parameters (RBCs, Hb, and Hct) between the two groups. Moreover, higher THI was associated with the increase in RT and RR of both HT and NHT buffaloes (Figure 1). Finally, four buffaloes for each group (HT group: HT039, HT059, HT773, and HT825; NHT group: NH043, NH076, NH161, and NH164) were selected and used for further analysis.

**Table 1:** Evaluation of physiological and blood parameters in HT and NHT buffaloes (mean ± SEM).

Parameters	HT	NHT
HSP70 (pg/mL)	454.26 ± 91 .15^a^	142.86 ± 41.35^b^
HSP90 (pg/mL)	3972.53 ± 668.49^a^	845.42 ± 236.96^b^
Cortisol (ng/mL)	251.64 ± 10.68^a^	121.46 ± 24.20^b^
RT (°C)	38.72 ± 0.10^a^	39.67 ± 0.06^b^
RR (breaths/min)	41.67 ± 4.43^a^	100.12 ± 3.48^b^
RBCs (10^6^/μL)	5.59 ± 0.19	5.29 ± 0.21
Hb (g/dL)	114.25 ± 4.52	104.75 ± 3.01
Hct (%)	32.28 ± 1.19	29.75 ± 1.06


*^a,b^The different superscripts between two groups show significant differences (*p* < 0.05).*

### mRNA and miRNA Expression Profiles Between HT and NHT

To examine and characterize mRNA and miRNA expression profiles that are related to heat stress, we performed RNA-Seq and miRNA-Seq analysis using blood samples from HT and NHT animals. For RNA-Seq data, a total of approximately 52.29 million raw reads were obtained from each sample. After quality control, we obtained an average of approximately 53.19 million and 50.86 million clean reads from HT and NHT, respectively, for further analysis (Supplementary Table S2). For the miRNA-Seq data, an approximate average of 149.40 million raw reads were generated from each sample, and an approximate total of 131.43 million and 147.58 million clean reads from HT and NHT groups were used for further analysis (Supplementary Table S3). Moreover, the sequence length distribution of each clean read ranged from 20 to 24 nt (Supplementary Figure S3).

**FIGURE 2:**
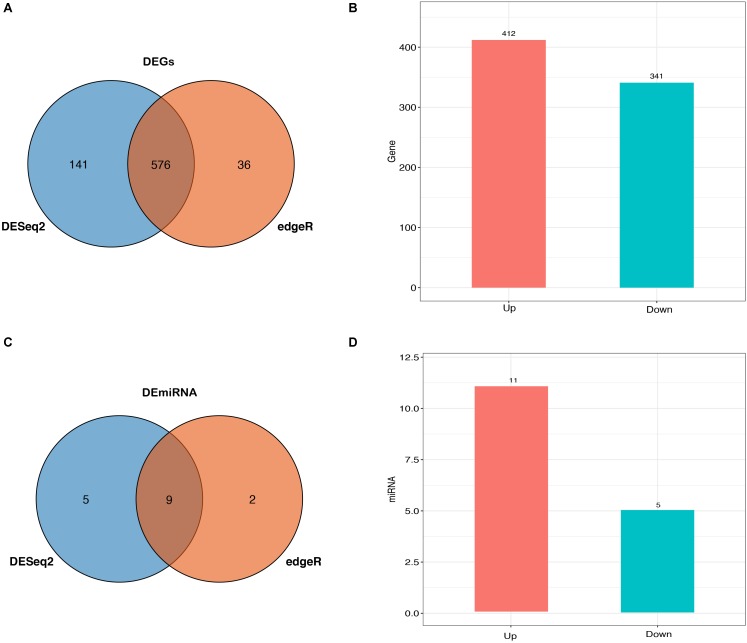
Expression profiles of buffaloes’ blood samples between HT and NHT. **(A)** Venn analysis of the identified DEGs from DEGSeq2 and edgeR. **(B)** Bar plots are showing the up- and down-regulated DEGs. **(C)** Venn analysis of the identified DEmiRNAs from DEGSeq2 and edgeR. **(D)** Bar plots are showing the up- and down-regulated DEmiRNAs.

Herein, a total of 33,696 mRNAs and 418 miRNAs in response to heat stress were detected between HT and NHT using RNA-Seq and miRNA-Seq data, of which 753 genes and 16 miRNAs were differentially expressed. Venn analysis of RNA-Seq data showed that 576 DEGs was shared between DESeq2 and edgeR method, 141 DEGs was the specific-DESeq2, and 36 DEGs was unique for the edgeR (Figure 2A). Compared with those in NHT, a total of 412 and 341 DEGs in HT were up-regulated and down-regulated, respectively (Figure 2B). The results of GO and KEGG analysis for the DEGs are listed in Supplementary Table S4. For miRNA-Seq data, a total of 16 DEmiRNAs were identified using the DESeq2 and edgeR, 9 of which were shared between two methods (Figure 2C). Among them, 11 up-regulated and 5 down-regulated DEmiRNAs were discovered in HT group compared to NHT group (Figure 2D). The characteristics of 16 DEmiRNAs between HT and NHT are displayed in Table 2.

**Table 2:** List of 16 DEmiRNAs between HT and NHT.

miRNA	Log2FC (HT/NHT)	*P*-value	Mature sequence	Precursor sequence
Bta-miR-1246	2.42	0.0053	aauggauuuuuggagcagg	caacauauuaaauggauuuuuggagcaggaaguuggaauagaggcuuucucagacaaaua
Bta-miR-1260b	2.02	0.0260	aucccaccacugccacca	aucccaccacugccaccacugcugcuacugcuccgcaggugcugcugguggugaugaug
				auaguccg
Bta-miR-2285az	4.29	0.0347	aaaauccgagugaacuuuuugg	aaaaguuugcuuggguuucccuguaagauguuauaggaaaauccgagugaacuuuuugg
Bta-miR-432	1.39	0.0437	ucuuggaguaggucauugggu	ucuuggaguaggucauuggguggauccuuuauuucccuaugugggccacuggauggcuccucca
				ugucu
Bta-miR-485	1.90	0.0384	gaggcuggccgugaugaauucg	agaggcuggccgugaugaauucgauucaucaaagcgagucauacacggcucu
				ccucucu
Novel-miR129	6.19	0.0113	gaaaagcucauucggguuuuu	gaaaagcucauucggguuuuuccaccugauguuacagaaaacccgauagaacuuuuugg
Novel-miR206	–8.70	2.29E-05	aaaaucugagugaaccuuuuga	aaaauguucauucaggguuuucugcaagacguuacaaaaaaucugagugaaccuuuuga
Novel-miR221	–10.96	5.33E-06	agaaagaggcacacccugguc	ccagggugugccuguuucuuucgugaccuugcuuuucuggugaagaaagaggcacacccugguc
Novel-miR231	5.67	0.0170	accacaguggcuaaguucu	accacaguggcuaaguucuauggcugauaugaccuucaucuugucuaucucucaacuuggucag
				uuauuggugg
Novel-miR246	–4.51	0.0306	auaaaguucguucggguuuu	auaaaguucguucggguuuucuuguaugacaucacagaaaaacuggaacaaacuuuuugg
Novel-miR353	–4.64	0.0183	agccuccucccggccccga	agccuccucccggccccgacuagaccaggcacucaccaaccaggccucugcuagagcugcccgg
				aggcaggggcuuc
Novel-miR357	4.29	0.0081	aggacccaggggcaagcagcuu	aucugcuuucuucuggaacugcaagaacccaggcaggacccaggggcaagcagcuu
Novel-miR359	5.27	0.0154	accucuucccugcucccccaga	aagggagguaggaggggcugggcggagcaugggggccaagcucaccgcccugaccucuucc
				cugcucccccaga
Novel-miR474	–5.61	0.0137	ucaaaaauucguucggguuuu	ucaaaaauucguucggguuuuuccacaacaucuuacagacaaacccaaaugaacuuuu
Novel-miR536	3.71	0.0284	uaccccugccuggacaccuggu	uaccccugccuggacaccugguagagcgugucucuucccgaggcagauggaccaaguuuccagg
				cagggggacc
Novel-miR669	1.91	0.0261	uuggcuuccccccuccccaga	caggggugggcagggaugcuaaggcuccguuuccccuuggcuuccccccuccccaga


### Co-expression Network Analysis and Modules Identification

**FIGURE 3:**
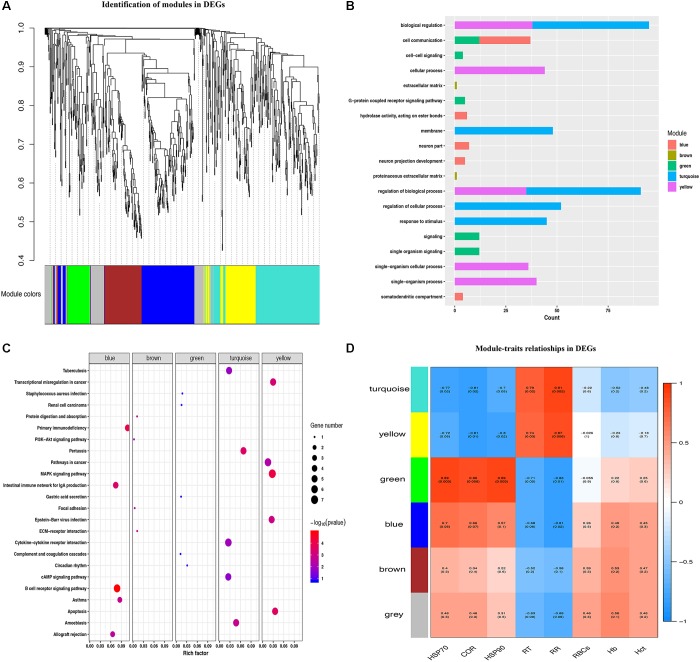
Identification of modules and functional annotation analysis for the module genes. **(A)** Module detection for DEGs. **(B)** GO analysis for the module genes. **(C)** KEGG enrichment analysis for module genes. **(D)** Module-trait relationships in DEGs.

In order to identify heat tolerance-associated modules and genes, WGCNA was performed on the identified DEGs. A total of 6 co-expression modules was identified (Figure 3A), with 69 DEGs clustered into the gray module. The turquoise module has the largest number of DEGs (220), while the green module has the smallest number of DEGs (68). The heat map for each module gene was showed in Supplementary Figure S4. All the modules were significantly enriched in the biological process term, displaying the strongest correlation with categories of cell communication (blue module), proteinaceous extracellular matrix (brown module), single organism signaling (green module), biological regulation (turquoise module), and cellular process (yellow module) (Figure 3B). KEGG enrichment analysis revealed that the modules with the largest gene number were the yellow, turquoise, and blue modules, which corresponds to the MAPK signaling pathway, Cytokine-cytokine receptor interaction, and B cell receptor signaling pathway (Figure 3C).

To better explore the module-trait significance, we performed association analysis between heat stress indexes and modules. As shown in Figure 3D, turquoise and yellow modules both showed significantly positive correlation with RT (*r* = 0.79, *P* = 0.02; *r* = 0.74, *P* = 0.03) and RR (*r* = 0.91, *P* = 0.002; *r* = 0.87, *P* = 0.006), and displayed significantly negative correlation with the plasma HSP70 (*r* = -0.77, *P* = 0.02; *r* = -0.72, *P* = 0.05), COR (*r* = -0.81, *P* = 0.02; *r* = -0.81, *P* = 0.01), and HSP90 (*r* = -0.7, *P* = 0.05; *r* = -0.8, *P* = 0.02) levels. The green module genes were found to be significantly positively correlated with plasma HSP70 (*r* = 0.89, *P* = 0.003), HSP90 (*r* = 0.89, *P* = 0.003) and COR (*r* = 0.86, *P* = 0.006) levels, and negatively correlated with RT (*r* = -0.71, *P* = 0.05) and RR (*r* = -0.83, *P* = 0.01). The blue module had a positive correlation with plasma HSP70 (*r* = 0.70, *P* = 0.05) levels and negative correlation with RR (*r* = -0.81, *P* = 0.02), respectively.

### Hub Genes Analysis

**FIGURE 4:**
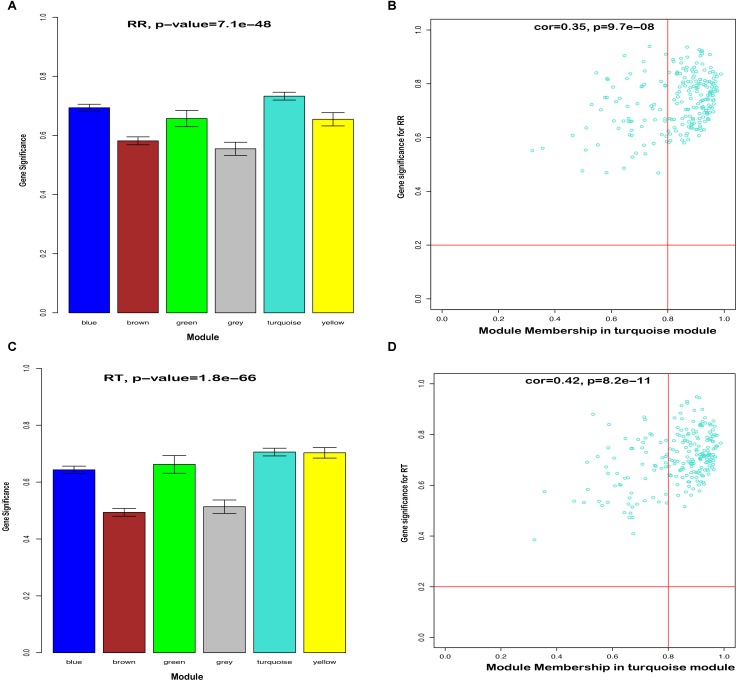
Hub genes detection in the turquoise module for RT and RR. **(A)** Histogram of correlation between module genes and RR. **(B)** Scatter plot of module eigengenes in the turquoise module for RR. **(C)** Histogram of correlation between module genes and RT. **(D)** Scatter plot of module eigengenes in the turquoise module for RT.

To further identify hub genes in the modules related to heat tolerance, we firstly qualified the relevance between eigenvalue of network modules and heat stress indexes. As shown in Supplementary Figure S5, the green module had highest gene significance for plasma HSP70, COR, and HSP90 levels than other modules (*P* < 0.05). For the RT and RR, the turquoise module exhibited a higher ability to indicate external traits accurately compared to other modules (Figure 4A,C). Our GO and KEGG analysis also showed that the turquoise module genes were significantly enriched in the Cytokine-cytokine receptor interaction, suggesting that these genes might be related to heat stress.

**FIGURE 5:**
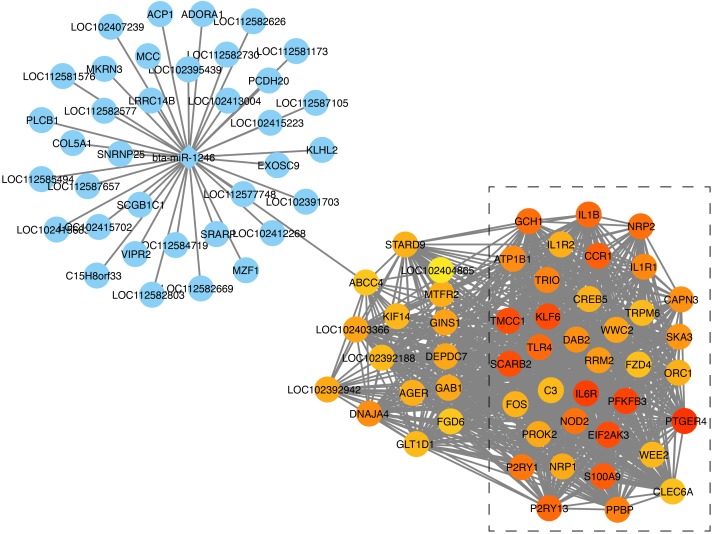
mRNA-miRNA network construction analysis. The genes in the dotted box were found within the PPI network; The circle represents genes; The rhombus represents miRNAs; The depth of gene’s color represents the its expression difference in RNA-Seq.

**FIGURE 6:**
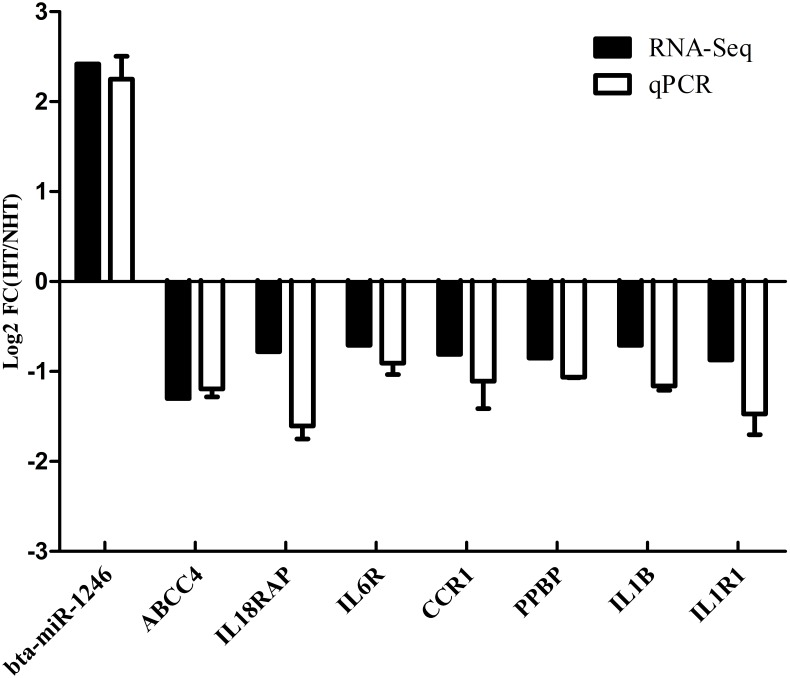
Validation of 6 hub genes by quantitative RT-PCR.

In the next step, we calculated correlation between GS and MM values of the turquoise module genes, aiming to identify hub genes in the interesting module. The scatter plots of GS related to RR and RT versus MM in the turquoise module are shown in Figure 4B,D. A total of 158 genes, as hub genes, highly associated with RR and RT traits in the turquoise module were identified, 35 of which were found within the PPI network using STRING analysis. The 35 DEGs can be treated as “real” hub genes (Table 3), and their PPI within dotted box can be visualized in Figure 5. Moreover, we found that bta-miR-1246 was targeted to the ABCC4 genes that can form an mRNA-miRNA network with the selected hub genes. Interestingly, a total of 6 hub genes were significantly enriched in the Cytokine-cytokine receptor interaction. Our finding suggests that the identified hub genes may be invovled in the biological process of RT and RR. Moreover, the results of qPCR showed that the expression level of the 6 hub genes displayed a similar tendency with that of the RNA-Seq (Figure 6). The expression levels of DEGs and DEmiRNAs were validated by qPCR (Supplementary Figure S6).

**Table 3:** Description of 6 hub gene in the Cytokine-cytokine receptor interaction pathway.

Gene	Full name	*P*-value	MM	GS for RT	GS for RR
IL18RAP	Interleukin 18 receptor accessory protein	0.0490	0.9102	0.6012	0.6075
IL6R	Interleukin 6 receptor	0.0222	0.9754	0.7540	0.7739
CCR1	C-C Motif chemokine receptor 1	0.0218	0.9136	0.7065	0.6424
PPBP	Pro-platelet basic protein	0.0163	0.9010	0.6308	0.7932
IL1B	Interleukin 1 beta	0.0467	0.9573	0.6690	0.7102
IL1R1	Interleukin 1 receptor type 1	0.0119	0.8833	0.6744	0.6471


## Discussion

Heat stress has a detrimental effect on animal growth and development, particularly the reduction of productive performance of these species (Aggarwal and Upadhyay, 2012; Wolfenson and Roth, 2018). Reducing the harm caused by heat stress has always been one of the biggest objectives of farmers and researchers. Measuring the heat stress index should be prioritized to provide some insights into the effect of heat stress on the productive performance of livestock. The RT and RR, for example, can serve as the physiological indicators of heat tolerance in cattle, which can quantify the changes in homeostasis by heat stress (Bianca, 1963). In the present study, our data showed that the physiological indicators including RT and RR in the HT group were significantly lower (*P* < 0.05) than that of the NHT group, which is similar to the results described by Garner et al. (2017). Subsequently, several studies reported that high temperature increase the expression levels of plasma HSP90 and HSP70 more markedly in HT than in NHT animals, such as cattle (Deb et al., 2014) and rodents (Jain et al., 2014). This is also supported by our findings. Moreover, our results showed that plasma COR levels in the HT buffalo group were higher than that of the NHT group, similar to previous studies of cattle (Hammond et al., 1996) and buffaloes (Shenhe et al., 2018). These findings also showed that the selection of buffalo samples in the present study is feasible and can be used for further analysis.

Transcriptome sequencing has become a powerful tool for identifying the candidate genes related to the complex traits or disease. Some candidate genes related to heat stress were identified in animals such as cattle (Srikanth et al., 2017), poultry (Coble et al., 2014), and swine (Yue et al., 2016). However, the changes of molecular mechanisms in animals under heat stress were complex, especially because information on the identification of candidate genes related to heat stress in buffaloes is limited. Therefore, to our knowledge, this is the first study to identify the candidate genes associated with heat tolerance in buffaloes using the transcriptome data. A total of 341 down-regulated and 412 up-regulated genes were identified between HT and NHT. The GO analysis showed that most DEGs were significantly enriched in the top 5 categories of cellular process, single-organism process, single-organism cellular process, biological regulation, and cytoplasm. Kapila et al. (2016) found that most heat responsive genes in buffaloes were significantly enriched in several biological processed including the cellular process, metabolic process, response to stimulus, and biological regulation, indicating that the identified DEGs in the present study were available. Of note, most of the DEGs were strongest enriched in the MAPK signaling pathway, suggesting that these DEGs may be involved in the regulation of heat tolerance in buffaloes. Moreover, a total of 16 DEmiRNAs was identified, 5 of which were the mature miRNAs and the remaining were the novel miRNAs. For them, a total of 11 and 5 DEmiRNAs were up-regulated and down-regulated between HT and NHT groups, respectively. A total of 87 target genes were predicted from the identified DEmiRNAs. The GO and KEGG analysis showed that most of the DEmiRNA target genes were significantly enriched in the negative regulation of cell migration and Natural killer cell mediated cytotoxicity, respectively. Notably, Jiang et al. (2004) reported that suppression of Natural killer cell mediated cytotoxicity was induced by cold stress, suggesting that the pathway might be related to heat stress.

To further explore the co-expression trend of DEGs, we performed the WGCNA analysis. They can be grouped into 5 modules, with the size ranging from 68 (green module) to 220 (turquoise module). Interestingly, four module genes (turquoise, yellow, blue, and green) were significantly correlated with physiological parameters (RT and RR) or ELSIA indexes (HSP70, HSP90, and COR). For the plasma HSP70, HSP90 and COR levels, the green module had the highest gene significance compare to other modules. Most genes of the green module were significantly enriched in the single organism signaling and cell communication. Notably, cell communication has also been reported to be involved in stress response (Lee et al., 2013). For the RT and RR, the turquoise module showed the highest gene significance, followed by the yellow module, and other modules. KEGG analysis revealed that most of the genes in the turquoise and yellow modules were significantly enriched in the Cytokine-cytokine receptor interaction and MAPK signaling pathway, respectively. Remarkably, Cytokine-cytokine receptor interaction pathway has also been demonstrated to be involved in stress response in humans (Schwaiger et al., 2016), renal MDCK I cells (Rasmussen et al., 2018), chicken thymus (Zhou et al., 2018), and rats (Li et al., 2013). This finding suggested that the turquoise module genes may be involved in the biological process of heat stress in buffaloes as well. Moreover, a total of 7 DEGs (including *LOC102409533, JUN, GADD45G, CD14, DUSP1, PLA2G4F*, and *HSPA1L*) in the yellow module were enriched in the MAPK signaling pathway. Previous studies have demonstrated that the MAPK signaling pathway was activated in response to many extra-cellular stimuli including UV radiation, osmotic shock and heat shock (Deng et al., 2007; Muthusamy and Piva, 2010; Srikanth et al., 2017). These results suggested that these DEGs can be considered candidate genes related to heat stress in buffaloes. Notably, some genes including *HSPA1* (Ortega et al., 2016), *DUSP1* (Kapila et al., 2016), and *CD14* (Selkirk et al., 2009) and *GADD45G* (Ying et al., 2005) were reported to be associated with heat stress. Meanwhile, blue module had a positive correlation with plasma HSP70 levels and negative correlation with RR, respectively. KEGG analysis revealed that most blue module genes were significantly enriched in the B cell receptor signaling pathway. Of note, Rothstein and Guo (2010) reported that B cell receptor signaling pathway was related to immune response, suggesting that the pathway might mediate the immune response induced by heat stress.

Identification of hub genes is critical for exploring heat-resistant mechanisms. Verma et al. (2000) highlighted RR and RT were the most sensitive indices of heat tolerance among all the physiological reactions studied. Consequently, in the present study, we focused on the identification of hub genes related to RR and RT. A total of 3 modules (turquoise, yellow, and green) were found to have the strongest correlations with RR and RT, while the turquoise module had the most significantly positive correlation with RR and RT. Our findings indicate that a total of 158 genes in the turquoise module can be considered as hub genes based on the selection criteria reported by Wang et al. (2017). Using this criterion, 35 genes were found within the PPI network using STRING analysis. Notably, most of these genes (*IL18RAP*, *IL6R*, *CCR1*, *PPBP*, *IL1B*, and *IL1R1*) were significantly enriched in the Cytokine-cytokine receptor interaction, suggesting that the 6 genes were treated as “real” hub genes. It should noted that *IL1B* and *IL1R1* were reported to be involved in heat stress and immune response (Slawinska et al., 2016; Rowland et al., 2018). Collier et al. (2008) reported that heat stress first activated *HSF1*, followed by increased the expression of heat shock proteins, reduction in fatty acid metabolism and endocrine system activation of the stress response, and finally the immune response system activation. Moreover, we found that bta-miR-1246 was targeted to the *ABCC4* gene that can form an mRNA-miRNA network with the selected hub genes. Interestingly, bta-miR-1246 has been demonstrated to be involved in heat stress response in cows (Zheng et al., 2014; Hu et al., 2018). The *ABCC4* gene, known as *MPR4*, was reported to be involved in cellular defense against oxidative stress (Ronaldson and Bendayan, 2008). Accordingly, these hub genes can serve as the candidate genes involved in heat stress and immune response in buffaloes, but further research is needed.

## Conclusion

We compared the heat stress indexes between HT and NHT buffaloes, indicating that HT buffaloes had a significantly lower RT and RR and displayed a higher plasma heat shock protein (HSP70 and HSP90) and COR levels compared to NHT buffaloes. A total of 753 DEGs and 16 DEmiRNAs were identified between HT and NHT buffaloes. Using the WGCNA analysis, a total of 5 modules were found to be associated with heat stress indexes. Importantly, six hub genes (*IL18RAP*, *IL6R*, *CCR1*, *PPBP*, *IL1B*, and *IL1R1*) in the turquoise module related to heat tolerance were identified, which is involved in the Cytokine-cytokine receptor interaction pathway. Finally, we constructed the mRNA-miRNA interaction network with the hub genes based on a combination analysis of RNA-Seq and miRNA-Seq. These findings will help in exploring underlying heat-resistant mechanisms in buffaloes.

## Data Availability

Publicly available datasets were analyzed in this study. This data can be found here: https://www.ncbi.nlm.nih.gov/bioproject/?term=PRJNA517372.

## Ethics Statement

Experimental animals, from Hubei Prime Cattle Husbandry Co., Ltd. (Jingmen, China) located at 113°25′ east longitude and 30°29′ north latitude were used in this study. All experimental designs and methods involving buffaloes in this study were approved by the Huazhong Agriculture University Animal Care and Use Committee (HZAUCA-2018-005).

## Author Contributions

SL, ZL, and JL collected the phenotype data and blood samples. SL and TY isolated plasma samples and RNA. TD created and carried out the analysis and interpreted the data. TD and SL wrote the manuscript. TD and LY developed the study and participated in its design and coordination. TD, YZ, AJ, AL, and GH reviewed the manuscript. All authors read and approved the manuscript.

## Conflict of Interest Statement

The authors declare that the research was conducted in the absence of any commercial or financial relationships that could be construed as a potential conflict of interest.

## Footnotes

1 http://www.bioinformatics.babraham.ac.uk/projects/fastqc/

2 http://www.mirbase.org/

3 https://string-db.org/

## Supplementary Material

FIGURE S1 The PCA plot for the selected buffaloes.

FIGURE S2 The pipelines of the bioinformatics data analysis.

FIGURE S3 The sequence length distribution of miRNAs.

FIGURE S4 The heat map for 5 module genes.

FIGURE S5 Diagram of correlation of module’s color and ELISA parameters.

FIGUR S6 Validation of RNA-Seq and miRNA-Seq results by quantitative RT-PCR.

TABLE S1 List of primers for mRNA and miRNA validation.

TABLE S2 Description of the RNA-Seq data in 8 buffalo blood tissues.

TABLE S3 Description of the miRNA-Seq data in 8 buffalo blood tissues.

TABLE S4 The GO and KEGG analysis of DEGs.
