# Supplementary figures and images for: Identifying Hub Genes for Heat Tolerance in Water Buffalo (Bubalus bubalis) Using Transcriptome Data
Source: Front Genet. 2019 Mar 13;10:209. doi: 10.3389/fgene.2019.00209 (PMC6424900; doi:10.3389/fgene.2019.00209)

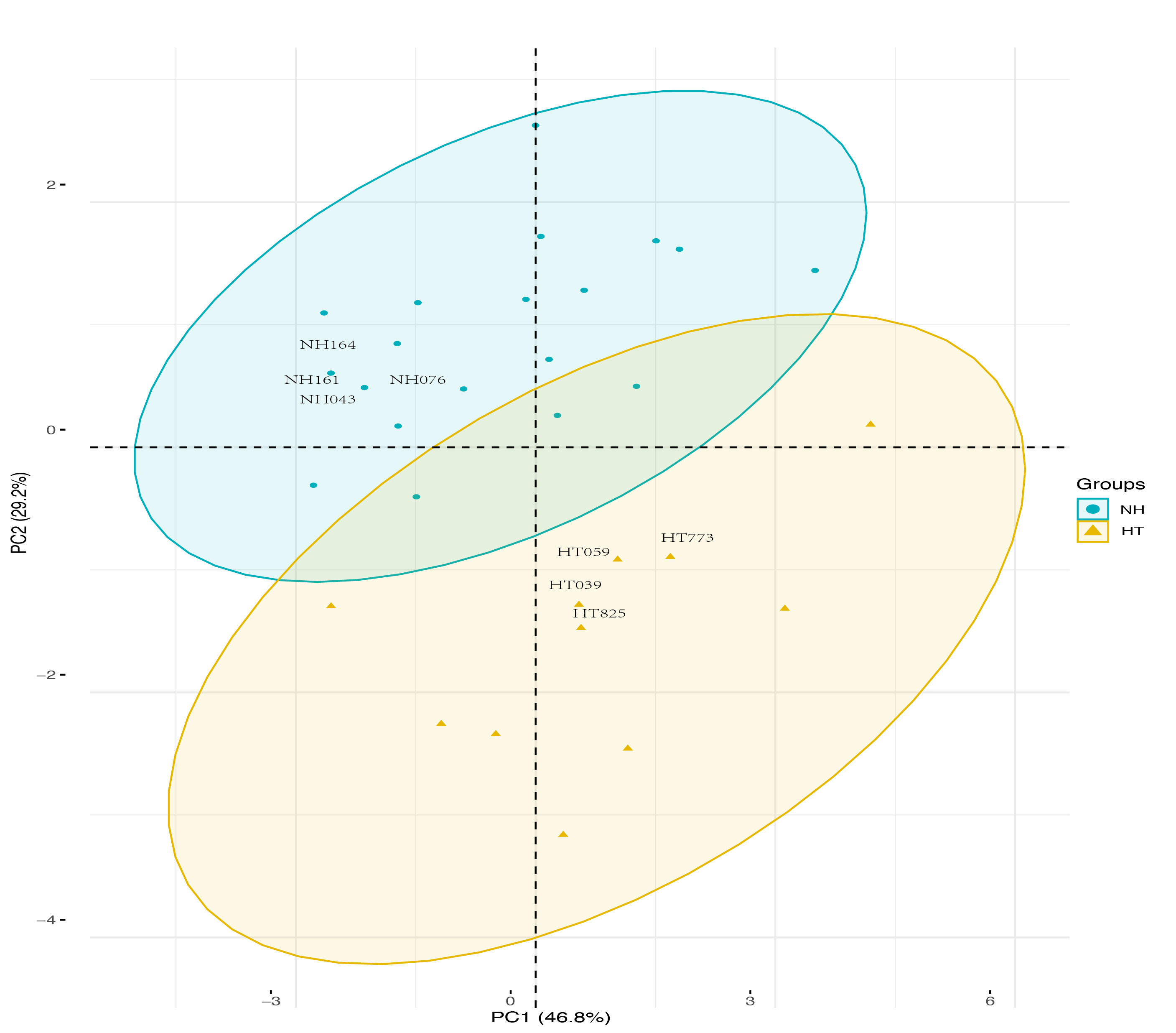

Supplement: FIGURE S1 — The PCA plot for the selected buffaloes. [file Image_1.JPEG]

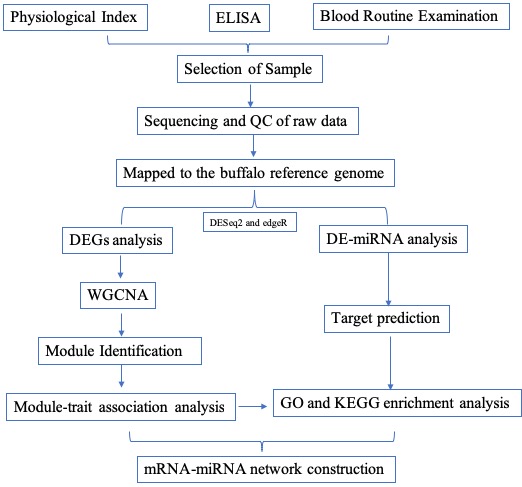

Supplement: FIGURE S2 — The pipelines of the bioinformatics data analysis. [file Image_2.JPEG]

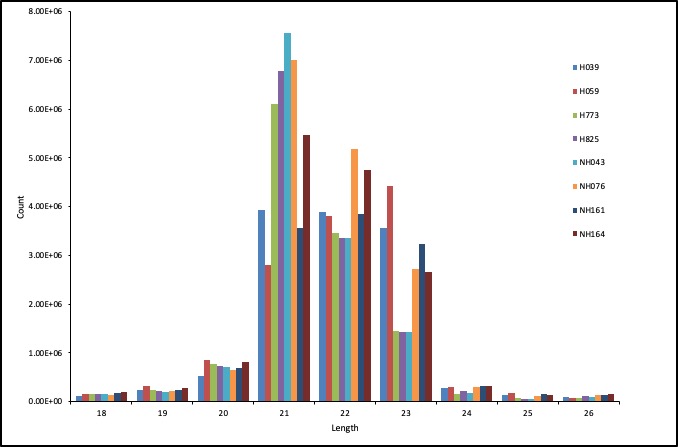

Supplement: FIGURE S3 — The sequence length distribution of miRNAs. [file Image_3.JPEG]

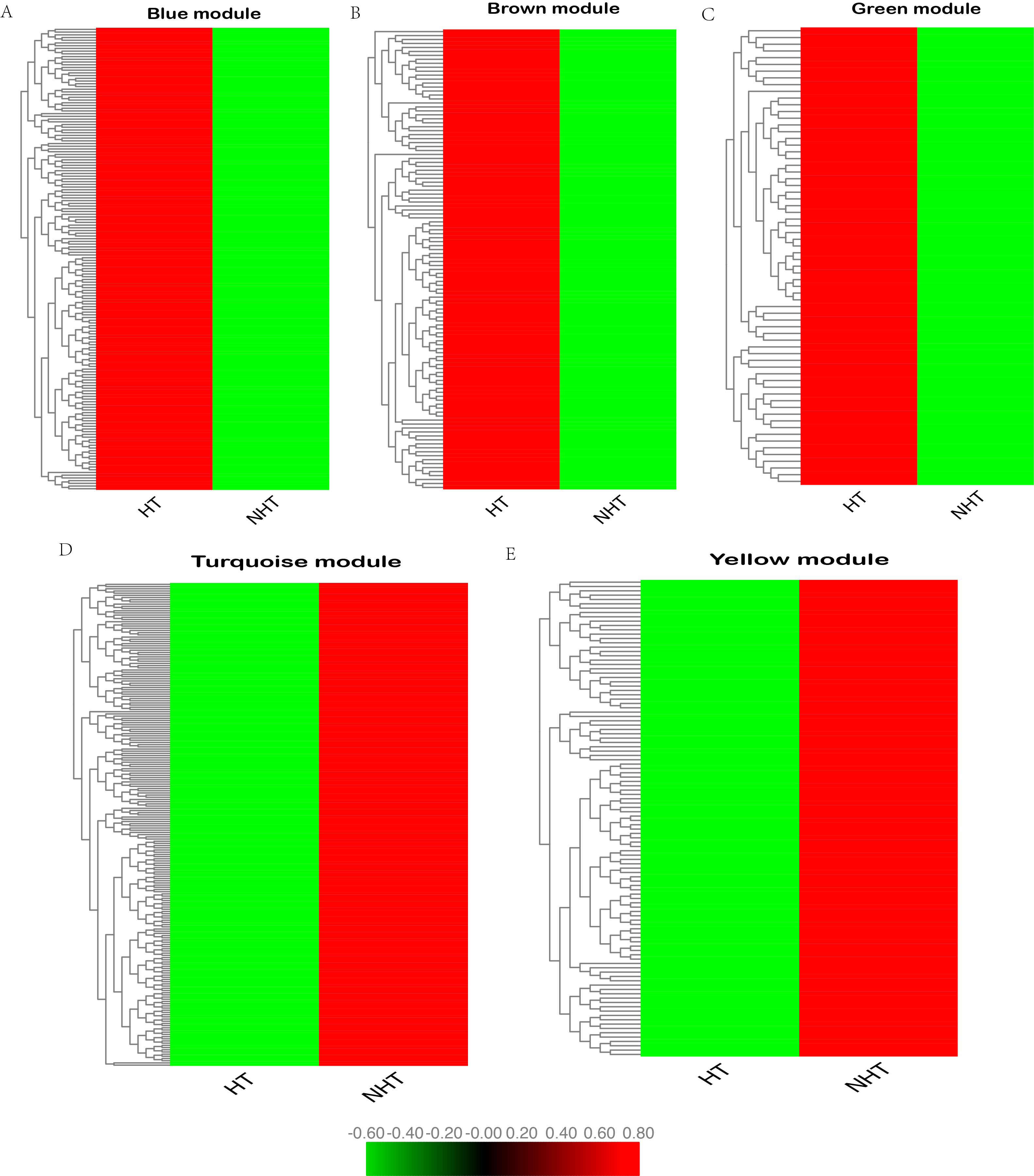

Supplement: FIGURE S4 — The heat map for 5 module genes. [file Image_4.JPEG]

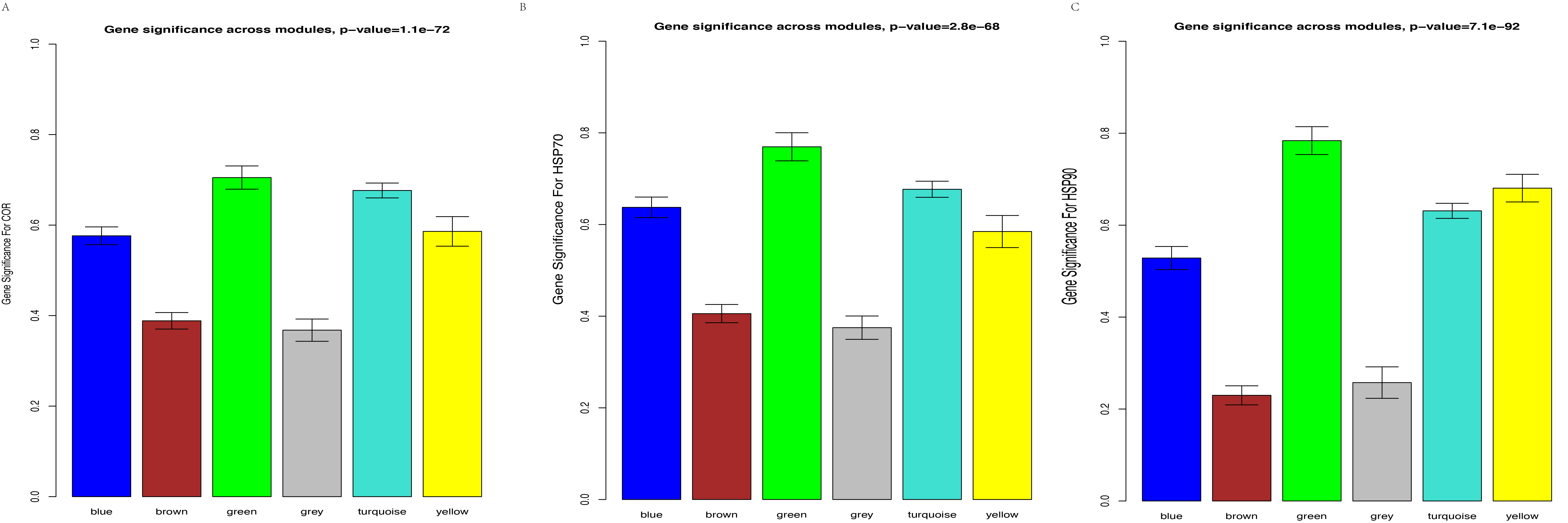

Supplement: FIGURE S5 — Diagram of correlation of module’s color and ELISA parameters. [file Image_5.JPEG]

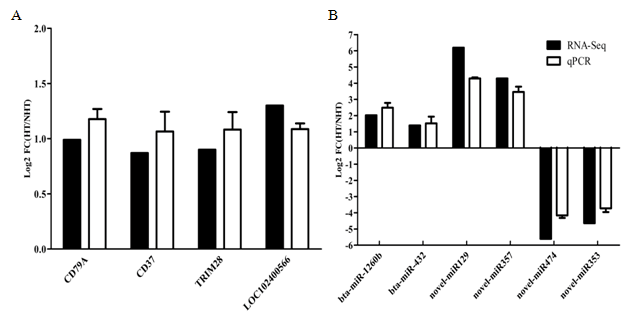

Supplement: FIGUR S6 — Validation of RNA-Seq and miRNA-Seq results by quantitative RT-PCR. [file Image_6.PNG]
